# Recombinant luteinizing hormone supplementation in women undergoing in vitro fertilization/ intracytoplasmic sperm injection with gonadotropin releasing hormone antagonist protocol: a systematic review and meta-analysis

**DOI:** 10.1186/1477-7827-12-109

**Published:** 2014-11-24

**Authors:** Yujing Xiong, Zhiqin Bu, Wei Dai, Meixiang Zhang, Xiao Bao, Yingpu Sun

**Affiliations:** Reproductive Medical Centre, the First Affiliated Hospital of Zhengzhou University, Zhengzhou, Henan Province China

**Keywords:** Recombinant FSH, Recombinant LH, Ovarian stimulation, Meta-analysis, Oral contraceptive pills

## Abstract

The objective of this meta-analysis is to assess the impact of LH supplementation in women undergoing in vitro fertilization/ intracytoplasmic sperm injection (IVF/ICSI) with gonadotropin releasing hormone (GnRH) antagonist protocol. No significant difference in outcomes between LH supplementation and r-FSH alone in women undergoing IVF/ICSI with GnRH antagonist protocol is currently present, and further studies are necessary for more solid conclusions on pregnancy likelihood to be drawn.

## Background

Compared to GnRH agonists which dominate in the area of assisted reproductive technology(ART) accounting for its essential role in circumventing the problem of a premature luteinizing hormone (LH) surge since the mid-1980s, GnRH antagonists generate a prompt suppression of gonadotrophin release, but do not cause the flare-up effect, by specifically blocking the GnRH receptors and ultimately induce a decrease in serum LH levels and a less pronounced decrease in FSH secretion [1]. However, it is unpredictable whether or not GnRH antagonists cause a decline in serum oestradiol during follicular recruitment which would result in an adverse effect on the pregnancy outcome [2]. On the other hand, GnRH antagonists tend to oversuppress endogenous LH if the dosage or timing of use was not appropriately controlled. As it is reported that endogenous low level of LH influences detrimentally both on the development of normal healthy follicles, because growing follicles become increasingly sensitive to and finally dependent on LH for their development [3], and on the endometrium after ovulation because sufficient LH is indispensable for the resumption of meiosis and for the production of progesterone. Therefore, it seems urgent for clinical doctors to add exogenous LH while GnRH antagonist protocol is applied to pituitary down-regulation in case of adverse effect on the pregnancy outcomes.

Nevertheless, there still has been no ultimate conclusion about the effect of r-LH supplementation to r-FSH in GnRH antagonist protocol on the pregnancy outcomes according to the recent studies. The issue on LH supplementation in women undergoing IVF/ICSI with GnRH antagonist for pituitary down-regulation has caused a heated debate around the world [4]. The studies by Sauer et al. (2004),Griesinger et al. (2005), Levi-Setti et al. (2006) did not demonstrate any beneficial effect of LH supplementation on the oocytes quality and the pregnancy outcomes [5–7], while two randomized trials have shown higher pregnancy rates among those receiving rLH with GnRH agonist protocol [8, 9]. It is noticeable that the meta-analysis published in 2007 and 2010 separately showed no advantage in combination of r-LH with r-FSH in women undergoing IVF/ICSI with GnRH antagonist protocol compared with r-FSH alone group [10, 11]. Given this background, the issue in this area warrants further research [12].

A recent systemic review and meta-analysis concluded that the combination of r-hLH with r-FSH stimulation enhanced the clinical pregnancy and implantation rates in patients aged ≥35 [13]. Similar results were reported in an open-label randomized controlled study by Bosch et al. (2011) which found that r-LH is beneficial in improving the implantation rate in women aged 36–39 years [14], although König et al. (2013) argued that the pretreatment with hormonal contraceptives before stimulation and the LH supplementation on stimulation day 1, while in his randomized controlled trial LH supplementation was given on stimulation day 6, might play an essential role in the discrepancy between two studies [15]. Until now, there has been no meta-analysis to review whether the LH supplementation benefits the advanced reproductive aged patients undergoing IVF/ICSI with GnRH antagonist protocol.

GnRH antagonist protocol depends on the occurrence of spontaneous menses, which is different from long GnRH agonist protocol in which ovarian stimulation can be initiated after pituitary desensitization has been achieved [16, 17]. Therefore, pretreatment with oral contraceptive pill (OCP) before stimulation was applied in order to prevent ovarian cysts, for the sake of synchronous follicular development and predictingtiming events in an IVF/ ICSI cycle regarding scheduling [18]. In the studies by Sauer et al. (2004), Levi-Setti et al. (2006), Bosch et al. (2011), the patients were pretreated with OCP and used the GnRH antagonist protocol for COH, but no special benefits was shown in r-LF + r-FSH group compared with the r-FSH only group [6, 7, 14]. Consequently, it is necessary to explore whether combination of r-LH with r-FSH for COH benefits the pregnancy outcomes in women undergoing IVF or ICSI-ET with GnRH antagonist protocol and oral contraceptive pills pretreatment by meta-analysis.

Based on the above considerations, the present meta-analysis was performed to answer the questions: (1) whether combination of r-LH with r-FSH for COH benefits the pregnancy outcomes in general women undergoing IVF/ICSI with GnRH antagonist protocol;(2) whether combination of r-LH with r-FSH for COH benefits the pregnancy outcomes in advanced reproductive aged women undergoing IVF/ICSI with GnRH antagonist protocol; (3) whether combination of r-LH with r-FSH for COH benefits the pregnancy outcomes in women undergoing IVF/ICSI with GnRH antagonist protocol and pretreated with oral contraceptive pills.

## Methods

### Systematic search and strategy

A systemic search of the relevant literature was performed without language limitation but restricted to randomized controlled trials (RCTs). We mainly explored MEDLINE, EMBASE, Web of science and Cochrane Library for the relevant studies about the effect of combination of r-LH with r-FSH for COH in patients undergoing IVF/ICSI with GnRH-antagonist protocol on IVF/ICSI outcomes. The following search strategy was used: ("luteinizing hormone" or "recombinant luteinizing hormone" or "lh" or "r-LH" or "hlh" or "recombinant lh" or "ovarian stimulation" or "recombinant FSH" or "lutropin alfa" or "recombinat human LH") AND ( "GnRH antagonist") AND (“assisted reproductive techniques” or “ART” or “IVF”or “ICSI” or “in vitro fertilization”or “intracytoplasmic sperm injections”) AND (“randomized controlled trial ” or “clinical trial” or “multicenter study” or “controlled study” or “double blind procedure” or “single blind procedure”).

### Inclusion and exclusion criteria

Inclusion criteria were RCTs that compared the effect of recombinant follicle-stimulating hormone (r-FSH) alone and combination with recombinant luteinizing hormone (r-LH) in women undergoing IVF/ICSI with GnRH antagonist protocol on IVF/ICSI outcomes. Exclusion criteria included failure to report appropriate randomization procedures, participants as poor responders, or outcomes unclear or inappropriate.

### Data extraction

Studies were screened by two reviewers (Y.X. and Z.B.) independently and any disagreement was solved unanimously by discussion. Firstly, all titles and abstracts from the databases were examined, but only those with the possibility of meeting the predefined criteria were kept for further evaluation. Secondly, final inclusion decisions were made on examination of the full manuscripts. If the published study was judged to contain insufficient information, study authors were contacted. The following data were recorded from each of the studies: methodologic (randomization method)was declared, number of patients included (rLH + rFSH/rFSH), inclusion criteria, ovarian stimulation protocol, Gn type and initial dosage (IU/d), Gn type and initial dosage (IU/d), rLH protocol, use of oral contraceptive pretreatment with and primary outcomes in each article.

### Outcome parameters

The main outcome measure chosen for the current meta-analysis was ongoing pregnancy per ET (defined as the presence of fetal heart activity on ultrasound at 12 weeks of gestation per ET) and clinical pregnancy per ET. The primary adverse effect was OHSS. Secondary outcome measures included days of stimulation, amount of r-FSH dose used, number of retrieved oocytes per oocyte retrieval, number of mature oocytes (metaphase II) per oocyte retrieval, fertilization rate, implantation rate, serum oestrodial on hCG day (pg/ml), serum progesterone on hCG day (ng/ml).

### Quantitative analysis

All results were combined for meta-analysis with Revman Software (Version 5, The Cochrane Collaboration, 2003). Continuous variables were expressed as weighted mean difference (WMD) with 95% confidence intervals (CIs). Dichotomous data for each unit of analysis were expressed as an odds ratio (OR) with 95% CIs. Heterogeneity was evaluated using the Q-test and I^2^-index values, and reported for each outcome as a P-value and percentage, respectively. Bias was assessed at the study level using a qualitative review assessing randomization, double blinding, and withdrawals and dropouts. In the absence of statistical heterogeneity a summary estimate of the odds ratio with a 95% was calculated in a fixed-effect model using the Peto modification of the Mantel- Haenszel method. In case of significant statistical heterogeneity, we performed sensitivity analysis using the random-effect model. A P-value <0.05 was considered statistically significant.

## Results

### Systematic review

**Figure 1:**
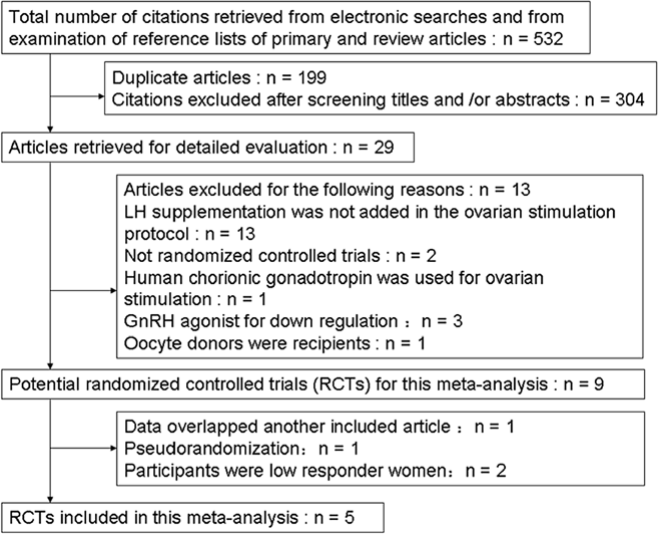
**Flow chart of the study selection process used for a systematic review and meta-analysis, undertaken to investigate the effect of recombinant human luteinizing hormone supplementation in women undergoing IVF/ICSI with antagonist protocol.**

The search strategy retrieved a total of 532 references, 29 full text articles were reviewed, and from these 5 trials met full inclusion criteria [5–7, 14, 15] and were included in the analysis with no disagreement noted between the reviewers responsible for study selection (Figure 1). Further details about these studies are provided in the Table 1. All the five trials were meta-analyzed to compare r-FSH combined with r-LH versus r-FSH alone in GnRH antagonist protocols in the general population. Two trials were meta-analyzed to compare r-FSH combined with r-LH versus r-FSH alone in GnRH antagonist protocols in advanced reproductive aged women [14, 15]. In addition, three trials were meta-analyzed to compare r-FSH combined with r-LH versus r-FSH alone in GnRH antagonist protocols in patients pretreated with oral contraceptive pills [6, 7, 14].

**Table 1:** **Characteristics of included studies**

Included RCTs	Method of randomization	Number of patients (rLHtrFSH/rFSH)	Gn type and initial dosage (IU/d)	rLH protocol	Pretreament	Primary outcomes
Sauer et al. 2004 [5]	Computer generated	21/21	r-hFSH 225	r-hLH, 150 IU on stimulation day 7–10	Oral contraceptive pretretment (0.15 mg desogestrel and 0.03 mg ethinyloestradiol)	Mean number of retrieved
						MII oocytes
Griesinger et al. 2005 [6]	Sealed envelop	61/65	r-hFSH 150	rLH, 75 IU on day 2 of the natural cycle	None	Number of days of gonadotropin treatment
Levi-Setti et al. 2006 [7]	Computer-generated list	20/20	r-hFSH 150	rLH, 75 IU when follicles reached the mean diameter of 14 and 15 mm	Oral contraceptive ((Minulet; Wyeth, Aprilia-Latinia, Italy))	Number of metaphase II oocytes retrieved
Bosch et al. 2011 [14]	Computer-generated list	Aged <35 years:190/190; aged 36 to 39 years: 170/170	Patients ≤ 35 years old: rFSH-alone group: rFSH 225 ;the rFSH + rLH group: rFSH 150; patients aged 36–39 years: rFSH-alone group: rFSH 300; rFSH + rLH group: rFSH 225	rLH, 75 IU on stimulation day 1	Oral contraceptive pill (0.030 mg ethinyl E2 and 3.0 mg drospirenone)	Implantation rate
König et al. 2013 [15]	Sealed envelopes	125/128	r-hFSH, 225 IU	rLH, 150 IU on stimulation day 6	None	Implantation rate; clinical pregnancy rate

### Meta-analysis

Combination of r -LH with r -FSH versus r -FSH alone for COH in general population undergoing IVF or ICSI-ET with GnRH antagonist protocol.

### Primary outcomes

#### Ongoing pregnancy per ET

**Figure 2:**
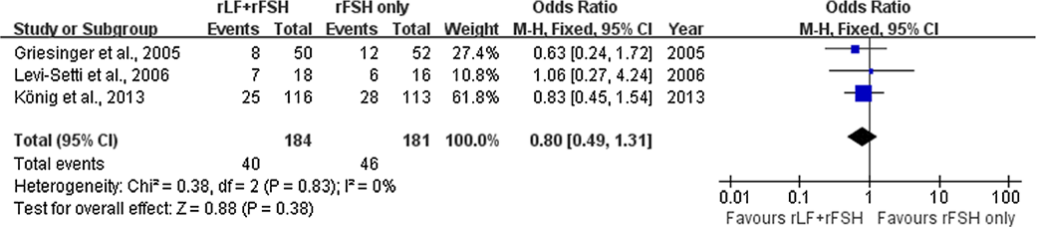
**Forest plot of ongoing pregnancy per ET with or without r-LH supplementation for COH in general population undergoing IVF or ICSI-ET with GnRH antagonist protocol.**

Three trials with a total of 365 embryo transfers provided data on the ongoing pregnancy per ET [5, 7, 15]. The pooled analysis with these three trials did not show differences between the r-LH supplementation group and the r-FSH alone group (three trials: OR 0.80; 95% CI 0.49 to 1.31) and there was no indication of statistical heterogeneity (Figure 2).

#### Clinical pregnancy per ET

**Figure 3:**
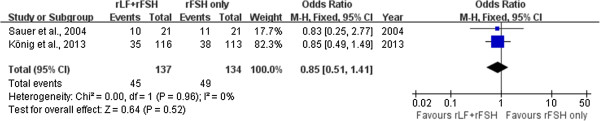
**Forest plot of clinical pregnancy per ET with or without r-LH supplementation for COH in general population undergoing IVF or ICSI-ET with GnRH antagonist protocol.**

Two trials with a total of 271 embryo transfers provided data on the clinical pregnancy per ET [6, 15]. The pooled analysis with these three trials did not show differences between the r-LH supplementation group and the r-FSH alone group (two trials: OR 0.90; 95% CI 0.65 to 1.42) and there was no indication of statistical heterogeneity (Figure 3).

### Incidence of ovarian hyperstimulation syndrome (OHSS)

**Figure 4:**
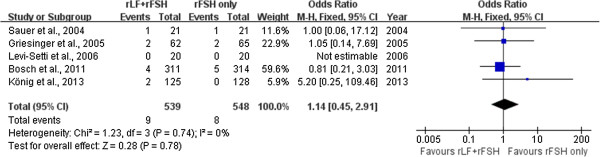
**Forest plot of incidence of OHSS with or without r-LH supplementation for COH in general population undergoing IVF or ICSI-ET with GnRH antagonist protocol.**

There was no evidence of a statistical difference in incidence of OHSS (five trials: OR 1.14, 95% CI 0.45 to 2.91) and there was no indication of statistical heterogeneity (Figure 4).

### Secondary outcomes

**Figure 5:**
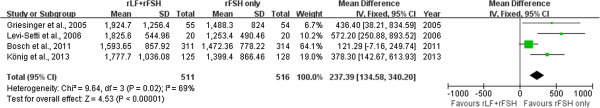
**Forest plot of serum oestrodial level on hCG day with or without r-LH supplementation for COH in general population undergoing IVF or ICSI-ET with GnRH antagonist protocol.**

**Figure 6:**
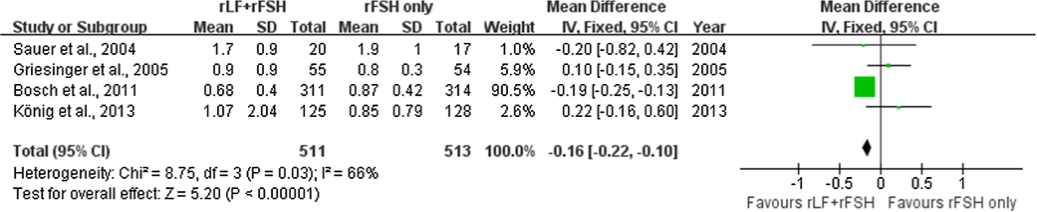
**Forest plot of serum progesterone level on hCG day with or without r-LH supplementation for COH in general population undergoing IVF or ICSI-ET with GnRH antagonist protocol.**

Four trials reported on serum progesterone level on hCG day [5, 7, 14, 15]. Pooling the data resulted in a significantly higher serum oestradiol level (WMD 237.39, 95% CI 134.58 to 340.20) (Figure 5) and lower serum progesterone level (WMD -0.16, 95% CI -0.22 to -0.10) in the r-LH supplementation group than in the r-FSH alone group (Figure 6).

The data from the trials was pooled separately and there was no evidence of a statistical difference in r-FSH total dose used per treatment cycle regarding r-FSH total dose used per treatment cycle (four trials: WMD -77.96, 95% CI -211.46 to 55.53), total days of stimulation per treatment cycle (four trials: WMD 0.20, 95% CI -0.37 to 0.76), number of retrieved oocytes per oocyte retrieval(four trials: WMD 0.58, 95% CI -1.27 to 0.1), number of mature oocytes (metaphase II) per oocyte retrieved(two trials: OR 0.88; 95% CI 0.66 to 1.17), fertilization rate(two trials: OR1.03; 95% CI 0.89 to 1.20) and implantation rate (three trials: OR 0.76; 95% CI 1.51 to 1.13).

Combination of r-LH with r-FSH versus r-FSH alone for COH in advanced reproductive aged women undergoing IVF or ICSI-ET with GnRH antagonist protocol r-FSH total dose used per treatment cycle.

### r-FSH total dose used per treatment cycle

**Figure 7:**
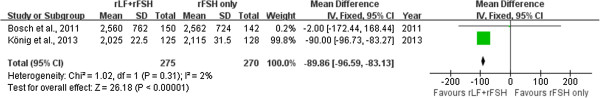
**Forest plot of rFSH total dose used per treatment cycle with or without r-LH supplementation for COH in advanced reproductive aged women undergoing IVF or ICSI-ET with GnRH antagonist protocol.**

Two trials reported data on r-FSH dose used per treatment cycle [5, 14]. There was a statistical difference in r-FSH total dose used per treatment cycle (two trials: WMD -89.86, 95% CI -96.59 to -83.13) (Figure 7).

### Serum oestrodial level on hCG day

**Figure 8:**
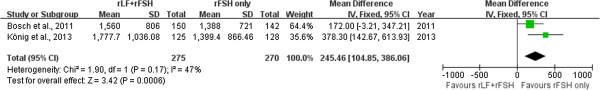
**Forest plot of serum oestrodial level on hCG day with or without r-LH supplementation for COH in advanced reproductive aged women undergoing IVF or ICSI-ET with GnRH antagonist protocol.**

Two trials reported on serum oestradiol level on hCG day [14, 15]. Pooling the data resulted in a significantly higher serum oestradiol level in the r-LH supplementation group than in the r-FSH alone group (WMD 245.46, 95% CI 104.85 to 386.06) (Figure 8).

### Number of retrieved oocytes per oocyte retrieval

**Figure 9:**
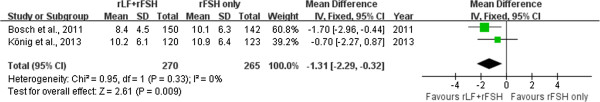
**Forest plot of number of retrieved oocytes per oocyte retrieval with or without r-LH supplementation for COH in advanced reproductive aged women undergoing IVF or ICSI-ET with GnRH antagonist protocol.**

Both of the two trials reported on the number of retrieved oocytes per oocyte retrieval [14, 15]. Pooling the data showed a significantly higher number of retrieved oocytes per oocyte retrieval in the r-FSH alone group (two trials: WMD -1.3, 95% CI -2.29 to -0.32) (Figure 9).

### Other outcomes

Pooling the data in the trials did not show a significant difference between the r-LH supplementation group and the r-FSH alone group regarding the total days of stimulation per treatment cycle (two trials: WMD -0.05, 95% CI -0.73 to 0.64), incidence of OHSS (three trials: OR 1.77, 95% CI 0.38 to 8.32), serum progesterone level on hCG day (WMD -0.04, 95% CI -0.46 to -0.38).

Combination of r-LH with r-FSH versus r-FSH alone for COH in women undergoing IVF or ICSI-ET with GnRH antagonist protocol and oral contraceptive pills pretreatment.

### Total days of stimulation per treatment cycle

**Figure 10:**
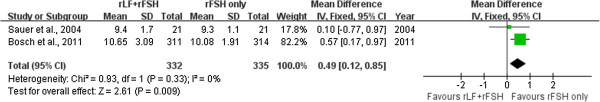
**Forest plot of total days of stimulation per treatment cycle with or without r-LH supplementation for COH in women undergoing IVF or ICSI-ET with GnRH antagonist protocol and oral contraceptive pills pretreatment.**

Two trials reported on the total days of stimulation per treatment cycle [6, 14]. Pooling the data showed a significantly higher total days of stimulation per treatment cycle in the r-LH supplementation group than in the r-FSH alone group (two trials: WMD 0.49, 95% CI 0.12 to 0.85) and there was no indication of statistical heterogeneity (Figure 10).

### Serum progesterone level on hCG day

**Figure 11:**
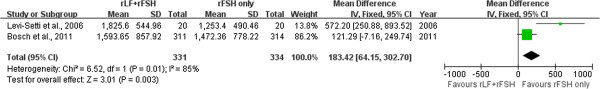
**Forest plot of serum progesterone level on hCG day with or without r-LH supplementation for COH in women undergoing IVF or ICSI-ET with GnRH antagonist protocol and oral contraceptive pills pretreatment.**

Two trials reported on serum oestradiol and progesterone level on hCG day [7, 14]. Pooling the data resulted in a significantly higher serum progesterone level in the r-FSH alone group than in the r-LH supplementation group (WMD -0.19, 95% CI -0.25 to -0.13) (Figure 11).

### Other outcomes

No evidence of a significant difference was found between the r-LH supplementation group and the r-FSH alone group regarding incidence of ovarian hyperstimulation syndrome (OHSS) (three trials: OR 0.84, 95% CI 0.25 to 2.78) , r-FSH total dose used per treatment cycle (two trials: WMD -211.90, 95% CI -319.99 to -103.82), serum oestrodial level on hCG day (WMD 321.71, 95% CI -117.44 to 760.86), number of retrieved oocytes per oocyte retrieval (two trials: WMD -0.69, 95% CI -1.52 to 0.13), r-FSH total dose used per treatment cycle (two trials: WMD -211.90, 95% CI -319.99 to -103.82).

## Discussion

Our systematic review and meta-analysis addressed the issue on the comparison of the outcomes between the combination of r-LH with r-FSH and r-FSH alone for COH in women undergoing IVF/ICSI with GnRH antagonist protocol and the comparisons in the subgroups of advanced reproductive aged women and women pretreated with oral contraceptive pills were also carried out.

Based on the “two-cell, two-gonadotropin” theory, the LH and FSH play a critical role in stimulating the two cellular components of ovary, which are theca cell and granulosa cell, leading to the production of ovarian steroids [19, 20]. At the earlier stage of follicular development, FSH is indispensable for follicular growth and the formation of estrogen by inducing the aromatase enzyme converting androgen to estradiol [21], while the androgen production from cholesterol is dependent on the stimulation of the theca cells by LH and FSH together [22]. Although FSH can induce follicular growth even without LH, there was identified that the follicles would have developmental deficiencies, following hCG administration [23], which suggested that the effect of LH on follicular development was probably not only due to providing androgen substrate for aromatization, but also exerting a direct effect on the stimulation and modulation of folliculogenesis [24]. It is noticeable that both the theca cell and granulosa cell produce significant amount of progesterone, which was converted into androgens under the influence of LH. Therefore, the LH supplementation resulted in the lower serum progesterone level. As is well established, increased exposure to progesterone can advance the endometrium, leading to asynchrony of embryo development to endometrial development and the reduction of implantation. Under this context, the LH supplementation may be beneficial for the serum oestradiol and progesterone level on the day of HCG administration.

As is predicted, our results suggested a beneficial effect of r-LH supplementation on ovarian stimulation in serum oestradiol and progesterone level on the day of HCG administration in general population. However, there was no evidence of beneficial effect in ongoing pregnancy per ET; clinical pregnancy per ET; incidence of OHSS; r-FSH total dose used per treatment cycle; total days of stimulation per treatment cycle; number of retrieved oocytes per oocyte retrieval; number of mature oocytes (metaphase II) per oocyte retrieval; fertilization rate; implantation rate, which was in accordance with the result of the meta-analysis by Monique H Mochtar et al. (2010) [3].

With regard to LH supplementation for the advanced reproductive aged women undergoing IVF or ICSI with GnRH antagonist protocol, different trials showed different results. The study by Bosch et al. (2011) obtained a significantly better implantation rate and a clinically better ongoing pregnancy rate among those patients aged 36 to 39. However, the study by König et al. 2013 showed no benefit of LH supplementation in controlled ovarian stimulation for IVF/ICSI with GnRH antagonists on pregnancy rates in patients of 35 years or older. Then we pooled the data from the two trials, showing a significantly higher serum oestradiol level and significantly lower r-FSH total dose used per treatment cycle found in the combination of r-LH with r-FSH compared to r-FSH alone for COH in advanced reproductive aged women undergoing IVF/ICSI with GnRH antagonist protocol. Although a significantly lower number of retrieved oocytes per oocyte retrieval was also found in the combination of r-LH with r-FSH group and the data of ongoing pregnancy or clinical pregnancy per ET were not available, we can not make the conclusion that LH supplementation was not beneficial for advanced reproductive aged women, since according to Bosch et al., the combination of r-LH with r-FSH group showed similar metaphase II oocytes and a better fertilization rate, thus suggesting that the oocytes obtained were of better quality, which would, in turn, lead to a higher implantation [14]. More trials and meta-analyses are needed to explore the role of LH supplementation played in advanced reproductive aged women.

Since oral contraceptive pills pretreatment is a convenient way for clinics to schedule oocyte retrievals, it will be more often applied by US clinics, although it is reported that oral contraceptive pills pretreatment diminishes the advantages of a GnRH antagonist protocol by extending the duration of treatment and the amount of FSH required to get to the same criteria of hCG [25], especially when stimulation is started immediately after OC withdrawal. This meta-analysis was not designed to detect a clinical relevant difference in ongoing pregnancy rate between pretreatment with or without oral contraceptive pills. Our results suggest a good effect of r-LH supplementation in ovarian stimulation in serum progesterone level on the day of HCG administration (WMD -0.19, 95% CI -0.25 to -0.13). Significantly lower serum progesterone level was observed in the combination of r-LH with r-FSH group compared to r-FSH alone group for COH in women undergoing IVF or ICSI with GnRH antagonist protocol and oral contraceptive pills pretreatment. This might be due to the fact that oral contraceptive pills pretreatment could have had an influence on the endocrine environment in the follicular phase by means of endogenous gonadotropin control [26] and FSH acts on granulosa cells to facilitate the conversion of cholesterol into P, which is transferred to the thecal cells to be converted into androgens under the action of LH, therefore LH supplementation lowered serum progesterone level [27]. Moreover, lower progesterone level, subsequently, increased endometrium receptivity. Under this context, LH supplementation may be the optimal option by increasing the beneficial effect of LH administration in this particular population. Our results also show a significantly higher total days of stimulation per treatment cycle (WMD 0.49, 95% CI 0.12 to 0.85) while significantly lower r-FSH total dose used per treatment cycle (WMD -211.90, 95% CI -319.99 to -103.82).

However, it has to be acknowledged that there still are some limitations in our meta-analysis. Firstly, the combined sample size of the five studies is still too small to confidentially detect a clinically relevant difference with regard to pregnancy likelihood between the two treatment modalities, especially when taking the subgroups into consideration, only the data from two or three trials were available for meta-analysis. Secondly, inclusion criteria of the selected trials were not as strict as possible, meaning that the inclusion criteria bias existed in the paper. For instance, when analyzing the outcomes of combination of r-LH with r-FSH comparing with r-FSH alone for COH in women undergoing IVF or ICSI with GnRH antagonist protocol in general population general population, all trials should be limited into those in which all the patients were pretreated with oral contraceptive pills or not, the initiation of LH supplementation was on the same stimulation day, and also ended on another same day, moreover, the initial dosage of FSH and LH was kept accordance with each trials. The last but not the least, the data of the trials was not all available for the meta-analysis, although the author was contacted if necessary, which resulted into that less trials were analyzed in the subgroup.

## Conclusions

To conclude, the present meta-analysis found no statistically significant differences in outcomes of pregnancy between the combination of r-LH with r-FSH group and r-FSH alone group for COH with GnRH antagonist protocol in general population, in advanced reproductive aged women and in women pretreated with oral contraceptive pills undergoing IVF/ICSI. More studies are necessary for more solid conclusions on pregnancy likelihood after combination of r-LH with r-FSH for COH in GnRH antagonist protocol to be drawn.

## Abbreviations

ART: Assisted reproductive technology
CI: Confidence intervals
COH: Controlled ovarian hyperstimulation
ET: Embryo transfer
GnRH: Gonadotropin-releasing hormone
hCG: human chorionic gonadotropin
ICSI: Intracytoplasmic sperm injection
IVF: In vitro fertilisation
OCP: oral contraceptive pill
OHSS: Ovarian hyperstimulation syndrome
OR: odds ratio
rFSH: Recombinant follicle-stimulating hormone
rLH: Recombinant luteinizing hormone
RTC: Randomized controlled trials
SD: standard deviation
WMD: weighted mean difference.
